# Microbiota and Autism: A Review on Oral and Gut Microbiome Analysis Through 16S rRNA Sequencing

**DOI:** 10.3390/biomedicines12122686

**Published:** 2024-11-25

**Authors:** Federico Anaclerio, Maria Minelli, Ivana Antonucci, Valentina Gatta, Liborio Stuppia

**Affiliations:** 1 Center for Advanced Studies and Technology (CAST), “G. d’Annunzio” University of Chieti-Pescara, 66100 Chieti, Italy; mariaminelli60@gmail.com (M.M.); i.antonucci@unich.it (I.A.); v.gatta@unich.it (V.G.); stuppia@unich.it (L.S.); 2 Department of Neurosciences, Imaging and Clinical Sciences, “G. d’Annunzio” University of Chieti-Pescara, 66100 Chieti, Italy; 3 Department of Medical Genetics, “G. d’Annunzio” University of Chieti-Pescara, 66100 Chieti, Italy; 4 Department of Psychological, Health and Territorial Sciences, “G. d’Annunzio” University of Chieti-Pescara, 66100 Chieti, Italy

**Keywords:** 16S rRNA, ASD, gut microbiota, microbiome, NGS, oral microbiota

## Abstract

Autism spectrum disorder (ASD) is a complex neurodevelopmental condition with multifactorial etiologies, including genetic, environmental, and microbiological factors. In recent years, increasing attention has been given to the role of the gut microbiota in ASD. Emerging evidence suggests that gut microbiota dysbiosis may influence the central nervous system through the gut-brain axis, potentially impacting behavior and neurodevelopment. The use of 16S rRNA gene sequencing has become a pivotal tool in profiling the microbial communities associated with ASD, offering valuable insights into bacterial diversity, composition, and potential functional roles. This review aims to provide a comprehensive analysis of current findings on the relationship between the gut and oral microbiota with ASD, and a particular focus on studies utilizing 16S rRNA sequencing. We will explore how gut microbiome alterations may contribute to ASD pathophysiology, discuss the limitations of existing research, and propose future directions for the integration of microbiome analysis in ASD diagnostics and treatment strategies. These findings underscore the potential role of microbiota in modulating ASD symptoms. The data suggest that specific bacterial taxa are consistently altered in ASD, which may have implications for understanding the gut-brain axis and its influence on neurodevelopment.

## 1. Introduction

Autism spectrum disorder (ASD) is a neurodevelopmental condition characterized by deficits in social communication, repetitive behaviors, and restricted interests. Its prevalence has increased significantly over the past two decades, now affecting approximately 1 in 100 children globally [1]. ASD is widely regarded as a multifactorial disorder, with contributions from both genetic predispositions and environmental influences. While extensive research has focused on identifying genetic mutations and environmental triggers, recent attention has shifted to the role of the microbiota, particularly in relation to the gut-brain axis, as a potential contributor to ASD pathogenesis [2]. The human microbiome is the collective term for the trillions of microorganisms that inhabit various niches of the body, including the skin, vaginal cavity, oral cavity, and gastrointestinal tract. When talking of the microbiome as a community, we generally mean all forms of microorganisms, including bacteria, viruses, and fungi, living together in our body [3,4]. The human microbiome is an extraordinary example of mutualism among organisms that, living together, benefit from each other. The main groups of microorganisms in the gut are *Bacteroides*, *Firmicutes*, and *Proteobacteria* [5]. Balance among them is essential; an imbalance, called dysbiosis, in fact, can affect gut health. A balanced microbial composition, where commensal and beneficial bacteria predominate over pathogenic species, is essential for maintaining host health and supporting immune function. Among these, the gut microbiota has received considerable attention for its profound impact on host metabolism, immune regulation, and even neurodevelopmental processes [6]. The composition and diversity of the gut microbiota can be influenced by several factors, such as genetics, diet, mode of delivery (vaginal vs. cesarean), [7] antibiotic use, and environmental exposures [8,9]. Dysbiosis, or an imbalance in the microbial community, has been implicated in various health conditions, including gastrointestinal disorders, metabolic syndrome, and neuropsychiatric diseases such as ASD [10]. A growing body of research has identified significant alterations in the oral and gut microbiota of individuals with ASD compared to neurotypical individuals, particularly in the gut and oral environments. This dysbiosis may contribute to both gastrointestinal symptoms, which are commonly observed in ASD, and the behavioral and cognitive symptoms characteristic of the disorder [11]. Gastrointestinal problems such as constipation, diarrhea, and abdominal pain are prevalent in individuals with ASD, and these issues are often linked to alterations in the gut microbiota. Moreover, the gut-brain axis, a bidirectional communication network between the gut and the Central Nervous System (CNS), is believed to play a critical role in influencing behavior, mood, and cognition through microbial metabolites, immune signaling, and the production of neurotransmitters [12,13]. The advent of high-throughput sequencing technologies, particularly 16S ribosomal RNA (rRNA) sequencing, has revolutionized our understanding of the microbiome by enabling comprehensive profiling of microbial communities in various body sites. 16S rRNA sequencing targets the 16S ribosomal RNA gene, which is highly conserved across bacterial species but contains hypervariable regions that allow for species-level identification [14]. This method has been widely used to study the composition and diversity of microbial communities in ASD, providing valuable insights into the microbial shifts that may be linked to the disorder. Several studies have reported consistent alterations in the gut microbiota of individuals with ASD, including reduced microbial diversity and shifts in the relative abundances of specific bacterial taxa, revealed using Next Generation Sequencing (NGS) technology. For example, decreases in beneficial bacteria such as *Bifidobacterium* and *Lactobacillus*, alongside increases in potentially pathogenic genera such as *Clostridium*, have been observed [15,16]. These microbial imbalances may lead to altered production of short-chain fatty acids (SCFAs), such as butyrate, propionate, and acetate, which are known to influence gut barrier integrity, immune function, and even brain function through the gut-brain axis [17,18]. The potential for these microbial alterations to impact neurodevelopment is supported by animal models of ASD, which demonstrate that manipulating the gut microbiota can influence behaviors relevant to autism, such as social interaction and stereotypes [19]. Beyond the gut, the oral microbiota is another important but less studied component of the human microbiome in the context of ASD. The oral cavity harbors a complex and dynamic microbial community that plays a key role in both oral and systemic health. Dysbiosis in the oral microbiota has been linked to various oral diseases, such as periodontitis and dental caries, as well as systemic conditions like cardiovascular disease and diabetes [20,21]. Alterations in the oral microbiota have also been observed in individuals with ASD, potentially contributing to both oral health problems and systemic inflammatory responses. Some studies suggest that microbial changes in the oral cavity may reflect or influence the gut microbiota due to the continuous microbial exchange along the gastrointestinal tract [22]. The relationship between oral and gut microbiota and ASD opens new avenues for potential therapeutic interventions. This review aims to explore the current understanding of the microbiota-gut-brain axis in ASD, emphasizing the mechanisms through which gut microbiota dysbiosis may contribute to ASD symptoms; moreover, the focus is to provide a comprehensive overview of how environmental, dietary, and microbial factors influence the MGB axis in the context of ASD. To provide a comprehensive summary of the current literature on the microbiota-gut-brain axis in ASD, we included peer-reviewed studies published between 2010 and 2023. Studies were selected based on relevance to ASD, the microbiome, and gut-brain signaling, with an emphasis on research that included quantitative data on doses of inflammatory agents, probiotics, and other interventions used in preclinical and clinical settings. Both animal models and human trials were considered to offer a balanced perspective. Inclusion criteria also prioritized studies that reported quantitative outcomes with statistical analyses, including sample sizes, dose information, and statistical significance where available. No specific selection criteria regarding patients’ ethnicity, age, sex, or dietary preferences was made.

## 2. The Gut-Brain Axis and ASD

The gut-brain axis refers to the bidirectional communication between the CNS and the gastrointestinal tract, integrating multiple pathways including neural, hormonal, immune, and metabolic routes. This axis involves neural, endocrine, and immune pathways [23] and it is increasingly recognized for its role in regulating mood, behavior, and cognitive function [9,24,25]. Its influence extends beyond normal physiological processes and is implicated in various neurodevelopmental and psychiatric disorders, including ASD [13]. In the context of ASD, it has been hypothesized that alterations in the gut microbiota may disrupt this communication, potentially leading to neurodevelopmental issues [26], suggesting a possible link between gut health and the disorder [27]. Emerging studies have shown that microbial dysbiosis in the gut may influence the CNS through mechanisms such as the production of neuroactive metabolites, immune modulation, and alterations in gut permeability [28].

The vagus nerve plays a critical role in the microbiota-gut-brain axis, serving as a primary bidirectional communication pathway between the gut microbiota and the CNS. Through vagal afferent fibers, signals generated by microbial metabolites in the gut, such as SCFAs and other neuroactive compounds, are transmitted to the brain, influencing neurodevelopmental and behavioral outcomes [29]. Recent studies have demonstrated that dysbiosis-induced changes in SCFA levels can alter vagal signaling, potentially leading to increased neuroinflammation and ASD-like behaviors [30]. Additionally, vagal pathways modulate immune responses by regulating cytokine production, thereby impacting neuroimmune interactions relevant to ASD pathophysiology.

**Figure 1:**
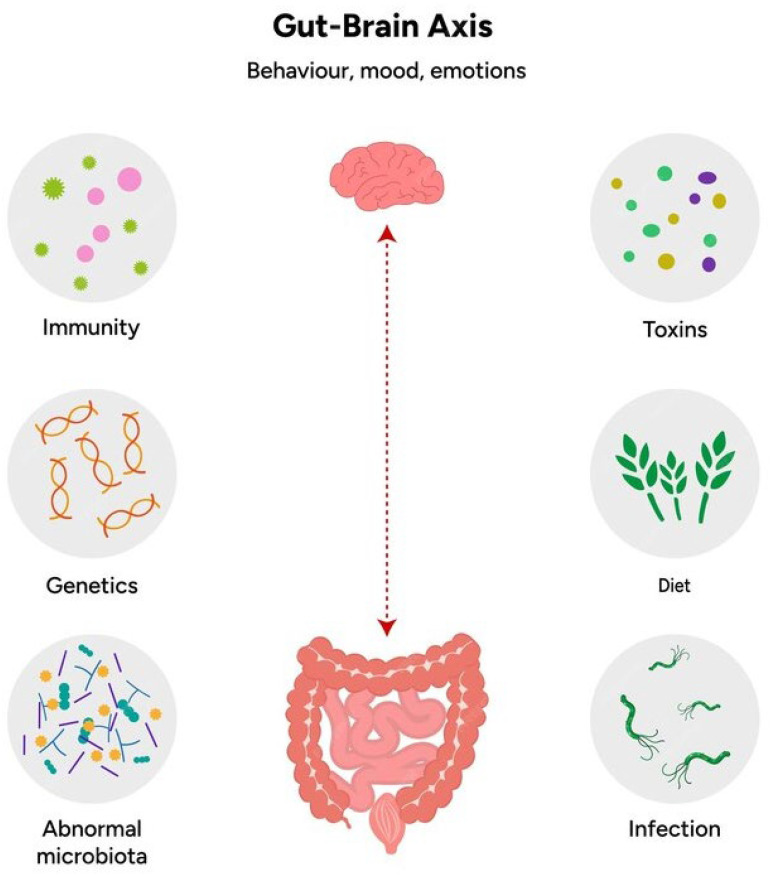
The gut-brain axis and its bidirectional communication.

The gut-brain axis encompasses several pathways that allow for continuous communication between the gut and the brain, and it is through this interplay that the gut microbiota is thought to influence neurodevelopment (as shown in Figure 1) [10]. The major communication routes include:

▪ Neural pathways: where the the vagus nerve plays a central role in transmitting signals from the gut to the brain, transporting sensory and microbial-derived information. In ASD, disruptions in vagal signaling due to microbial imbalances may contribute to altered brain function and behavior. In addition, the gastrointestinal tract possesses an intrinsic embedded nervous system, the Enteric Nervous System (ENS), which consists of a very large number of neurons (more than one hundred million in humans, corresponding, roughly, to the number of neurons in the spinal cord), collected in numerous bundles of efferent and afferent fibers that connect gut and brain bidirectionally through the vagus nerve. The ENS, also known as the “second brain”, is made up of internal intestinal neurons and glial cells that innervate several laminae proper to the intramuscular mucosa. This intrinsic network of neurons allows the gastrointestinal tract to partially maintain its function without any input from the CNS, while the ENS can receive sensory input from the Autonomic Nervous System (ANS) and transmit information to it.
▪ Immune system modulation: a significant portion of the body’s immune cells reside in the gut-associated lymphoid tissue. Dysbiosis, or an imbalance in gut microbiota, can lead to immune dysregulation, chronic inflammation, and the release of pro-inflammatory cytokines such as interleukin-6 (IL-6) and tumor necrosis factor-alpha (TNF-α). These cytokines can cross the blood-brain barrier (BBB) and influence neuroinflammation, which is commonly observed in ASD.
▪ Endocrine pathways: the gut microbiota influences the production of several hormones and neurotransmitters that affect the CNS. For example, gut bacteria produce SCFAs such as butyrate, propionate, and acetate, which have neuroactive properties. Dysbiosis in ASD patients has been associated with altered SCFA levels, which can impact brain development and function.
▪ Microbial metabolites: the gut microbiota produces a wide range of metabolites, including SCFAs and neurotransmitters such as serotonin and gamma-aminobutyric acid (GABA). These metabolites can modulate the brain through systemic circulation, affecting cognitive function, mood, and behavior. Dysbiosis can disrupt the production of these key metabolites, exacerbating symptoms of ASD [28].

Research indicates that children with ASD exhibit significant differences in their gut microbiota composition compared to neurotypical children. Several studies have identified a reduction in beneficial bacterial taxa such as *Bifidobacterium* and *Prevotella,* alongside an increase in potentially pathogenic bacteria like *Clostridium* and *Desulfovibrio*. Studies have indicated lower levels of the genus Bifidobacterium, which may play a protective role in autism due to its anti-inflammatory effects. Additionally, reductions have been observed in *Prevotella*, *Coprococcus*, and *Veillonellaceae*, microorganisms essential for carbohydrate digestion and fermentation. Conversely, certain bacterial groups—such as *Lactobacillus*, *Clostridium*, *Desulfovibrio*, *Caloramator*, *Alistipes*, *Sarcina*, *Akkermansia*, *Sutterellaceae*, and *Enterobacteriaceae*—are found in higher abundance among children with ASD and their siblings compared to healthy controls. *Desulfovibrio*, a common anaerobic bacillus known for its resistance to antibiotics like cephalosporins, is particularly prevalent in individuals with ASD [29]. Several studies have reported an overgrowth of *Clostridium* within the *Firmicutes* phylum in children with autism, with a particularly high presence of *Clostridium histolyticum* and *Clostridium perfringens* in stool samples. Members of the *Clostridiaceae* family can produce certain metabolites—such as phenols, p-cresol, and indole derivatives—that may be harmful to humans. These metabolites have been found in greater concentrations in fecal samples from children with autism spectrum disorder (ASD). Notably, recent studies suggested that glyphosate (GLY), an environmental pesticide, might contribute to autism’s pathogenesis by promoting the growth of toxin-producing *Clostridia*. Indeed, *Clostridium perfringens* and *Clostridium botulinum* show a high resistance to GLY, unlike beneficial bacteria such as *Bifidobacterium* and *Lactobacillus*. Consequently, environmental exposure to GLY could negatively impact the gut-brain axis, potentially playing a role in autism development by disrupting the microbiome and increasing *Clostridium* toxin production [30,31]. These alterations may contribute to the gastrointestinal disturbances commonly seen in ASD, which in turn may aggravate core ASD symptoms through gut-brain communication pathways. For instance, *Clostridium* species produce high levels of propionate, an SCFA that can have neurotoxic effects at elevated concentrations. Animal studies suggest that excess propionate can induce behavioral changes reminiscent of autism, including hyperactivity, social withdrawal, and repetitive behaviors. Furthermore, *Desulfovibrio* species are known to produce hydrogen sulfide, which can disrupt gut barrier integrity and promote systemic inflammation, further linking gut dysbiosis to neurodevelopmental issues. SCFAs, produced by the fermentation of dietary fibers by gut bacteria, have a significant influence on gut health and neurodevelopment [18]. Butyrate, propionate, and acetate are the most studied SCFAs in the context of ASD. Butyrate has anti-inflammatory properties and plays a key role in maintaining gut barrier integrity and modulating immune responses. However, excessive levels of propionate, often seen in ASD, have been linked to neurobehavioral abnormalities. Butyrate’s role in neurodevelopment is complex. It acts as a histone deacetylase inhibitor, influencing gene expression in the brain and promoting neuronal plasticity. Reduced butyrate production due to dysbiosis may impair these processes, contributing to ASD [18]. Alterations in gut permeability, commonly referred to as “leaky gut”, have been implicated in ASD. A compromised intestinal barrier allows for the translocation of bacteria and their metabolites, including lipopolysaccharides (LPS), into the bloodstream. This breach can trigger systemic inflammation and affect the blood brain barrier, which may lead to neuroinflammation and neuronal damage. Studies show that children with ASD often have increased intestinal permeability, which is closely associated with gut dysbiosis. LPS, a component of the cell wall of Gram-negative bacteria, has been shown to activate immune responses that affect both peripheral and central inflammation. In animal models, LPS administration leads to ASD-like behaviors, suggesting that gut-derived immune activation may play a role in the pathogenesis of ASD [2]. Another consideration about gut-brain axis is the possible impact of multi-organ toxicity that can influence the bidirectional communication. Environmental toxins, including heavy metals, pesticides, and other pollutants, have been shown to disrupt the microbiota-gut-brain (MGB) axis, intensifying neuroinflammatory processes and potentially exacerbating symptoms in ASD. These toxins often compromise gut barrier integrity, a condition commonly referred to as “leaky gut”, allowing microbial metabolites and endotoxins such as lipopolysaccharides (LPS) to translocate into the systemic circulation. Once in the bloodstream, LPS and other pro-inflammatory molecules can cross the blood-brain barrier (BBB) and trigger immune responses within the CNS, fueling a cascade of neuroinflammatory events [32,33]. The vagus nerve serves as a key pathway in the transmission of these inflammatory signals from the gut to the brain, contributing to heightened neuroinflammation in individuals exposed to environmental toxins, influencing microbial composition and enhancing the prevalence of pathogenic bacteria with the increase of LPS production. This change in microbial balance exacerbates gut permeability, facilitating the entry of inflammatory agents into systemic circulation and their eventual impact on brain health [34]. These findings underscore the importance of considering environmental and multi-organ toxicity factors when evaluating the MGB axis’s role in ASD.

Moreover, various environmental and dietary risk factors have been associated with the onset of ASD. For example, a maternal high-fat diet during pregnancy has been shown to alter the microbiota of newborns and may be linked to ASD risk in humans. Additionally, formula-fed infants tend to have higher levels of *Clostridium difficile* in their gut microbiota. Even brief courses of antibiotic treatment can cause persistent shifts in the gut microbiome in both humans and animal models. Studies indicate that children exposed to antibiotics within the first three years of life develop distinct gut microbiome profiles. In particular, prolonged use of macrolide antibiotics is associated with alterations in the gut microbiota and may be linked to higher risks of obesity and asthma in children [31].

## 3. 16S rRNA Sequencing in Microbiome Studies

rRNA gene sequencing has become a cornerstone of microbiome research, providing critical insights into the composition and diversity of microbial communities in various biological environments. The 16S rRNA gene, present in all prokaryotes, contains both highly conserved and variable regions, making it ideal for distinguishing between different bacterial taxa [35,36]. Sequencing the 16S rRNA gene enables researchers to analyze complex microbial populations, identifying bacteria at the genus and sometimes even at the species level without the need for traditional culturing techniques, which can be limiting due to the fastidious nature of some microbes [37,38]. The methodology of 16S rRNA sequencing involves amplifying the gene from DNA extracted from samples—whether fecal, oral, or other biological specimens—using specific primers that target conserved regions of the gene. These amplified sequences are then subjected to high-throughput sequencing platforms, such as Illumina or Ion Torrent, to generate massive amounts of data [39,40]. Once sequenced, the data are processed through bioinformatic pipelines that compare the sequences against reference databases (e.g., Greengenes, SILVA, or RDP) to assign taxonomic classifications [41]. In a 2024 study by Minelli et al. [42], with the use of NGS technology and 16S rRNA methodology, the authors, starting from a buccal swab, revelead an infection of *Spirochaete* phylum, in particular the *Treponema* genus, in a patient evaluated genetically due to dementia and late-onset dyskinesia. Generally, the *Spirochaete* phylum and the different species of *Spirochaetes* have been associated with many neurodegenerative diseases and other pathologies. The recognition of the *Spirochaetes* phylum in the oral microbiota of these patients is of incredible interest due to its potential role in neuroinflammatory processes. From a clinical view, this finding suggests that oral health and the composition of the microbiome should be considered as potential factors in the treatment and management of patients with neurodegenerative diseases. In the context of ASD, 16S rRNA sequencing has been instrumental in revealing microbial dysbiosis in both the gut and oral microbiota of affected individuals. Studies utilizing this technique have shown consistent alterations in microbial diversity, with a reduction in beneficial taxa like *Bifidobacterium* and *Prevotella* and an overrepresentation of potentially harmful bacteria such as *Clostridium* and *Desulfovibrio* [43,44]. These microbial changes may contribute to the gastrointestinal symptoms commonly observed in ASD patients, such as constipation, diarrhea, and abdominal pain, which in turn might exacerbate core ASD symptoms [28,45]. Moreover, 16S rRNA sequencing allows researchers to study the functional potential of the microbiota indirectly. By comparing microbiota profiles between ASD and neurotypical individuals, scientists have observed differences in microbial pathways associated with the production of SCFAs, neurotransmitters, and metabolites that influence immune function and gut permeability [18], regarding the role of the microbiota in modulating neurodevelopment and behavior through the gut-brain axis [46,47,48]. As the precision and cost-efficiency of 16S rRNA sequencing improve, it will remain a vital tool for understanding the intricate relationship between the microbiome and ASD. Recent research has increasingly highlighted the link between specific microbiome profiles and ASD symptomatology (as shown in Table 1). For instance, Zuffa S et al. [49] conducted a comprehensive analysis using 16S rRNA sequencing, identifying an increased abundance of bacterial taxa such as *Prevotella* and *Bacteroides* in the gut microbiota of ASD patients. These microbial profiles were strongly correlated with symptom severity, including behaviors commonly associated with ASD, such as social withdrawal and repetitive actions. The study also explored the role of these bacterial alterations in promoting neuroinflammation, positing that the observed shifts in microbiota composition could exacerbate inflammatory pathways via immune signaling across the MGB axis, potentially through vagal nerve activation. The findings suggest that therapeutic strategies aimed at modulating the microbiota—such as probiotics or fecal microbiota transplants—could help in reducing these neuroinflammatory responses and alleviating ASD symptoms by restoring a more balanced microbial environment [50].

### Characteristics of 16S rRNA Methodology

**Figure 2:**
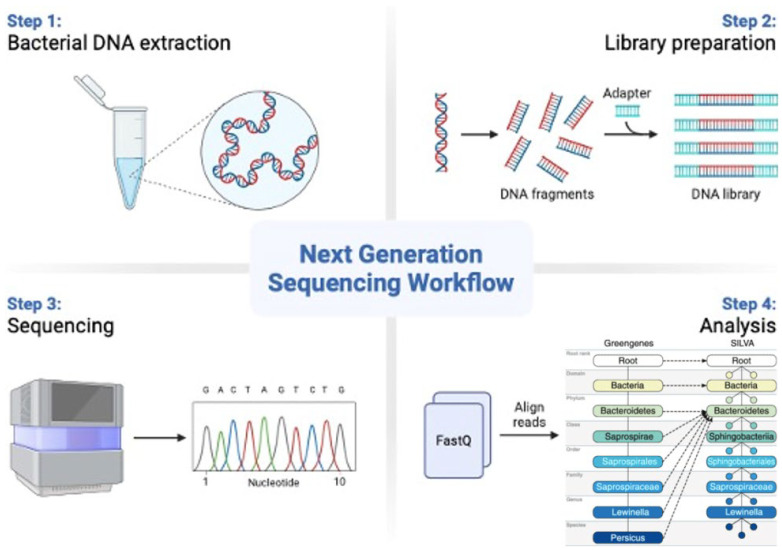
Illustrates the workflow of 16S rRNA sequencing, from sample preparation to taxonomic analysis.

The 16S rRNA sequencing technique has become a cornerstone in microbiome research due to its efficiency in profiling bacterial communities. The method targets the 16S ribosomal RNA gene, which contains both highly conserved and hypervariable regions that allow for the classification of bacteria at the genus level and, in some cases, at the species level. For each region, there are different primers designed to amplify that specific fragment. The process typically involves bacterial DNA extraction from samples, PCR amplification of the 16S rRNA gene’s hypervariable regions, high-throughput sequencing, and bioinformatics analysis to map the sequences against reference databases, such as SILVA or Greengenes, for taxonomic identification (as shown in Figure 2) [54,55].

However, while 16S rRNA sequencing is valuable for identifying bacterial composition, it has significant limitations. It does not capture the full range of microbial diversity, excluding non-bacterial organisms like viruses and fungi, and it provides limited information on functional genes, which limits insight into the metabolic capabilities of the microbiome. To address these limitations, more comprehensive approaches—such as metagenomics, which sequences all genetic material in a sample, including non-bacterial genomes—allow for a broader understanding of the microbial ecosystem.

## 4. Gut Microbiota and ASD

The gut microbiota refers to the vast community of microorganisms residing in the gastrointestinal tract. It plays a pivotal role in digestion, nutrient absorption, and the synthesis of essential vitamins. Additionally, the gut microbiota is integral to immune system function, protecting against pathogens and maintaining gut barrier integrity. Major bacterial phyla in the gut include *Firmicutes*, *Bacteroidetes*, *Actinobacteria*, and *Proteobacteria* [56,57]. A balanced gut microbiota contributes to overall health by regulating metabolic processes and modulating inflammatory responses. Dysbiosis, or microbial imbalance, can lead to a range of health issues, including inflammatory bowel diseases (IBD), obesity, and metabolic syndrome. Factors such as diet, antibiotic use, and lifestyle can significantly impact the composition and function of the gut microbiota [58,59]. Several studies have demonstrated that the gut microbiota in individuals with ASD is distinct from that of neurotypical individuals. For instance, some research has revealed a reduction in bacterial diversity in children with ASD, a finding that is commonly associated with chronic diseases [43]. Moreover, studies have identified alterations in specific bacterial genera, such as an increase in *Clostridium* and a decrease in *Bifidobacterium* and *Prevotella*, which are believed to play a role in maintaining gut health and regulating the immune system [45]. SCFAs, particularly propionate, and other metabolites produced by intestinal bacteria may directly influence ASD-related behaviors by affecting specific biological pathways. For instance, propionate has been shown to impact neurodevelopment through pathways that alter neurotransmitter balance, neuroinflammation, and mitochondrial function, which are associated with ASD phenotypes. These effects on brain physiology suggest a more direct role of bacterial metabolites in modulating ASD-related behavioral outcomes. The presence of microbial dysbiosis in ASD has led to the hypothesis that gut microbiota may contribute to ASD symptoms through the production of microbial metabolites, such as SCFAs like butyrate, acetate, and propionate [18,46]. Some studies have even suggested that higher levels of propionate-producing bacteria in the gut may contribute to behavioral abnormalities typical of ASD [60,61]. 

## 5. Oral Microbiota and ASD

**Figure 3:**
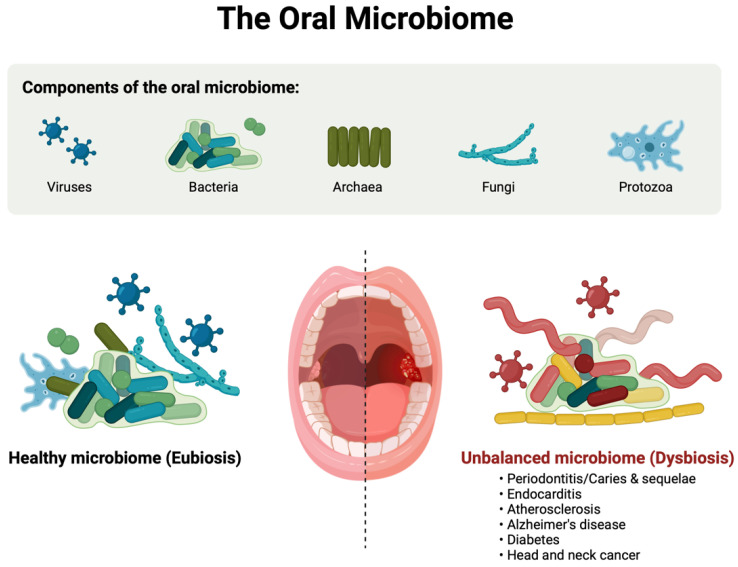
The composition of an oral microbiota and the differences between eubiosis and dysbiosis, which is linked to various disorders.

While much of the research on the microbiome in ASD has focused on the gut, there is growing interest in the role of the oral microbiota. The oral cavity hosts a complex and diverse microbial community, composed of over 700 microbial species, including bacteria, viruses, fungi, and archaea, that can influence both oral and systemic health [62]. This microbial community resides in different regions of the oral cavity, including the tongue, teeth, gums, palate, and cheeks, each of which harbors distinct microbial populations. The oral microbiota plays a fundamental role in maintaining oral homeostasis by interacting with the host’s immune system [63]. A situation of dysbiosis (as shown in Figure 3) [21] in the oral microbiota has been linked to various conditions, including periodontitis, cardiovascular disease, diabetes, and neurodegenerative disorders like Alzheimer’s disease [64,65]. The major phyla in oral microbiota are *Firmicutes*, *Bacteroidetes*, and *Proteobacteria*. Key bacterial genera in the oral microbiota include *Streptococcus*, *Porphyromonas*, *Fusobacterium*, and *Actinomyces*, which interact in a dynamic manner to form biofilms on the teeth and mucosal surfaces [66,67]. In the context of ASD, research is still in its early stages, but some studies have suggested that alterations in the oral microbiota may contribute to the disorder, either through direct interactions with the brain or via systemic inflammatory responses [68]. Recent findings using 16S rRNA sequencing have shown differences in the oral microbiota of individuals with ASD compared to neurotypical controls [22]. For example, one study found an increased abundance of *Porphyromonas* and *Prevotella* species in the oral cavities of children with ASD, both of which are known to be associated with inflammation [69]. These bacterial species are commonly associated with oral infections, such as gingivitis and periodontitis, and their overrepresentation in ASD patients suggests a potential link between oral inflammation and the broader symptomatology of ASD. One potential mechanism through which the oral microbiota may influence ASD is the production of neuroactive metabolites. Oral bacteria produce neuroactive metabolites, such as SCFAs, which may influence brain function. These metabolites can enter systemic circulation through the oral mucosa or gastrointestinal tract after swallowing. Once in the bloodstream, some of these compounds may cross the blood-brain barrier directly, while others may influence the brain indirectly by triggering immune responses or modulating systemic inflammation. These pathways provide potential mechanisms by which metabolites from the oral cavity can impact neurophysiological processes related to behavior. Certain oral bacteria are capable of producing metabolites such butyrate and propionate, which are known to have profound effects on both the gut and the brain [70]. SCFAs can cross the blood-brain barrier and influence brain function, particurarly in areas related to social behavior and cognitive function. Dysbiosis in the oral microbiota could lead to an overproduction of harmful metabolites or a reduction in protective compounds like butyrate, thus contributing to the neurodevelopmental aspects of ASD [71]. These alterations in the oral microbiota may contribute to gastrointestinal symptoms commonly observed in ASD, such as constipation and diarrhea, which in turn could affect behavior and cognitive function. These findings suggest that the oral microbiota, like the gut microbiota, may play a role in ASD through immune modulation and the production of bioactive metabolites [72].

## 6. The Microbiota-Immune System Interaction

Another crucial aspect of the gut-brain axis involves the interaction between the microbiome and the immune system. The gut is home to a significant portion of the body’s immune cells, and the microbiota plays a key role in regulating immune function [73]. Dysregulation of the immune system has been implicated in the development of ASD, with evidence suggesting that both the adaptive and innate immune systems may be involved [74]. Microbial dysbiosis can lead to chronic low-grade inflammation, which in turn may affect brain development and function [75]. Children with ASD have been found to exhibit elevated levels of pro-inflammatory cytokines, both systemically and in the brain, further supporting the notion that immune dysregulation may contribute to the disorder [76]. Alterations in the gut microbiota, such as a reduction in beneficial bacteria and an increase in pro-inflammatory species, may exacerbate this immune dysfunction, potentially influencing the severity of ASD symptoms [77].

## 7. Therapeutic Strategies for Patients with ASD and Altered Microbiota

Given the emerging evidence linking oral and gut microbiota alterations to ASD and the potential role of the dysbiosis that could play a role in neurodevelopmental disorders, therapeutic strategies aimed at modulating the microbiota have garnered significant interest. These approaches include dietary interventions, probiotics, prebiotics, fecal microbiota transplantation (FMT), and the use of antibiotics. Each of these strategies aims to restore a healthy balance of gut microbes and, in turn, alleviate some of the gastrointestinal and neurobehavioral symptoms associated with ASD [37,78].

### 7.1. Probiotics and Prebiotics

**Figure 4:**
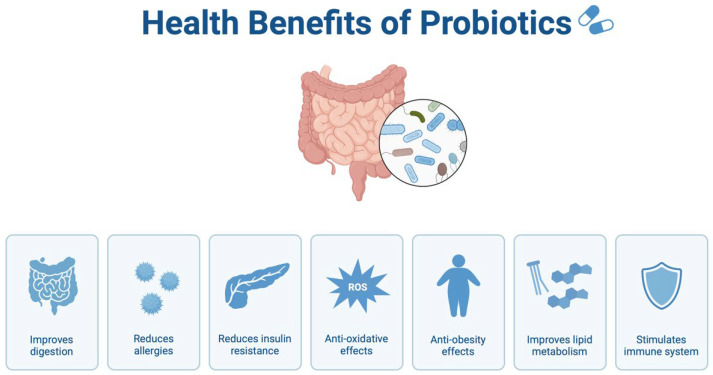
The benefits of the use of probiotics on human health.

Probiotics, which are live microorganisms that confer health benefits when administered in adequate amounts, have shown potential in modulating gut microbiota and improving symptoms in ASD patients. Studies have reported that the administration of specific probiotic strains, such as *Lactobacillus* and *Bifidobacterium*, can restore the balance of gut flora and reduce gastrointestinal issues (as shown in Figure 4) [79,80]. Probiotic supplementation has also been associated with improved behavioral outcomes, such as reductions in irritability and hyperactivity, possibly through the modulation of the gut-brain axis and the production of neuroactive compounds like serotonin [81]. Prebiotics, on the other hand, are non-digestible food components that promote the growth of beneficial bacteria in the gut. Common prebiotics include inulin, fructo-oligosaccharides (FOS), and galacto-oligosaccharides (GOS). These compounds selectively stimulate the growth of beneficial microbes, such as *Bifidobacterium* and *Lactobacillus*, thereby enhancing the overall health of the gut microbiota [82,83]. Some studies have suggested that prebiotic supplementation can improve both gastrointestinal and behavioral symptoms in children with ASD [84]. A study by Fattorusso et al. [31] reported the primary clinical trials performed on the effects of probiotics on children with ASD, during which they demonstrate both the advantages and the limits. Clinical trials investigating probiotic interventions in children with ASD have reported varying dosages and outcomes using different beneficial microrganisms. For example, a clinical trial by Kang et al. [51] administered a daily dose of *Lactobacillus rhamnosus* (10^10^ CFU) to 60 children with ASD, resulting in a significant reduction in gastrointestinal symptoms and behavioral issues (*p* < 0.05). In addition, in preclinical studies, the use of inflammatory agents in animal models of ASD has provided insights into dose-dependent effects on neuroinflammation. For instance, mice treated with lipopolysaccharide (LPS) at doses of 0.5 mg/kg showed increased ASD-like behaviors and elevated levels of cytokines IL-6 and TNF-α, with statistical significance (*p* < 0.01). These findings underscore the potential influence of specific dosages on inflammatory responses relevant to ASD pathophysiology. Although more research is needed, combining probiotics and prebiotics (synbiotics) offers a promising avenue for therapeutic intervention in ASD.

### 7.2. Dietary Interventions

Dietary approaches to managing ASD symptoms focus on altering the intake of food components that may exacerbate gut dysbiosis and neurobehavioral symptoms. One commonly used dietary intervention is the gluten-free, casein-free (GFCF) diet. Gluten and casein are proteins found in wheat and dairy, respectively, and it is hypothesized that these proteins, when improperly digested, produce peptides that can cross the blood-brain barrier and affect behavior in individuals with ASD [85,86]. While evidence supporting the effectiveness of the GFCF diet is mixed, some studies have reported improvements in gastrointestinal and behavioral symptoms following its implementation [87]. Another dietary intervention involves the ketogenic diet, a high-fat, low-carbohydrate diet traditionally used to treat epilepsy. The ketogenic diet alters the composition of the gut microbiota and increases the production of ketone bodies, which may have neuroprotective effects. Preliminary studies in ASD suggest that the ketogenic diet may reduce core autism symptoms and improve social communication [88]. However, the long-term effects and feasibility of this diet in ASD patients require further investigation.

### 7.3. Fecal Microbiota Transplantation

FMT is an innovative therapy that involves transferring fecal matter from a healthy donor to a patient in order to restore a balanced microbiota. Although FMT is still in its experimental stages for ASD, early studies have shown promising results. One groundbreaking study reported that children with ASD who underwent FMT experienced significant improvements in both gastrointestinal and behavioral symptoms (reduction of stereotypical behaviors and improvement in social interaction), with some effects persisting for up to two years post-treatment [51,89]. FMT has the potential to directly address microbial dysbiosis by introducing a diverse and balanced microbial community into the gut, thereby normalizing the gut-brain axis and behavioral symptoms [89,90,91,92]. By introducing a more diverse and balanced microbiota, FMT may influence this axis, helping to regulate neurodevelopmental processes that are disrupted in ASD. Furthermore, the introduction of beneficial microbes like *Bifidobacterium* and *Prevotella*, which are often lacking in ASD patients, can lead to enhanced production of SCFAs that positively influence brain function and immune responses. Several clinical trials have supported the therapeutic potential of FMT for ASD. A pioneering study conducted by Kang et al. (2017) showed that after 10 weeks of microbiota transfer therapy (MTT), children with ASD experienced significant reductions in both gastrointestinal (58%) and core behavioral symptoms (45%), with these improvements continuing for up to two years [51]. In another open-label study, researchers found that FMT improved gut barrier function and reduced systemic inflammation, two factors believed to play a critical role in ASD pathogenesis [93]. Despite the promise of FMT, challenges remain, including concerns about long-term safety, standardization of donor selection, and the potential for unintended consequences, such as the transmission of pathogens [51]. Nonetheless, as more research is conducted, FMT may become a viable therapeutic option for ASD patients with altered microbiota.

### 7.4. Emerging Biomarkers and Novel Therapeutic Approaches in ASD

Recent advances in bioinformatics have opened pathways to identifying and analyzing novel biomarkers with high potential for ASD diagnostics and treatment. The study of biomarkers such as microRNAs (miRNA), circulating free RNA (cfRNA), DNA methylation patterns, and extracellular vesicles (EVs), including exosomes, is now being supported by advanced bioinformatics tools that facilitate real-time data analysis, enabling precise disease forecasting and personalized interventions. miRNAs and cfRNA have emerged as promising biomarkers due to their stability in biofluids and their regulatory roles in gene expression [94,95,96]. For instance, altered levels of specific miRNAs in blood samples have been associated with ASD severity and symptomatology. These miRNAs, detectable through sequencing and bioinformatics platforms, are potential candidates for non-invasive diagnostics and offer insights into ASD-related gene regulatory networks [97]. Studies suggest that certain miRNA profiles can distinguish between ASD and neurotypical individuals with high accuracy, providing a foundation for real-time, accessible diagnostics. Moreover, EVs, especially exosomes, play crucial roles in cell-to-cell communication and have shown potential as biomarkers for ASD. Exosomes carry proteins, RNA, and other molecules that reflect the state of their cells of origin, including neurons and immune cells [98]. By isolating and analyzing exosomes from patient blood or cerebrospinal fluid, researchers can gain insights into neuroinflammatory states and neural development pathways associated with ASD. Bioinformatics tools are essential for analyzing the complex molecular cargo of exosomes, allowing for the identification of ASD-specific protein and RNA markers. Proteins and enzymes implicated in immune modulation, oxidative stress, and neurodevelopment have also been identified as biomarkers in ASD. Advances in bioinformatics enable large-scale proteomic analyses, allowing for the identification of ASD-specific proteins that could serve as therapeutic targets [99]. For example, increased levels of pro-inflammatory cytokines, detectable through proteomic analysis, have been correlated with ASD symptoms, supporting their potential role as biomarkers and targets for anti-inflammatory treatments. The identification of these biomarkers not only aids in diagnosis but also supports the development of personalized treatments. By targeting specific pathways associated with the identified biomarkers (e.g., miRNA-mediated gene regulation or exosome-based delivery of therapeutic agents), bioinformatics-driven approaches can facilitate tailored interventions that address the unique molecular profile of each ASD patient. These advancements in biomarker discovery and analysis tools are paving the way for personalized medicine in ASD, enabling early diagnosis, targeted treatment, and potentially improved clinical outcomes.

## 8. AI-Enhanced Bioinformatics and Multi-Omics Integration in ASD Research

The integration of multi-omics data—encompassing genomics, transcriptomics, metabolomics, and microbiomics—has the potential to revolutionize our understanding of ASD by providing a comprehensive view of the molecular and microbial influences on neurodevelopment. However, analyzing these vast datasets poses significant challenges due to data complexity, volume, and the need to extract clinically relevant insights. Recent advancements in Artificial intelligence (AI)-enhanced bioinformatics tools offer promising solutions to these challenges. Machine learning (ML) algorithms, for instance, can process high-dimensional data to identify patterns and biomarkers across multiple omics layers. Specific tools such as deep learning models and neural networks are increasingly applied to predict ASD-related outcomes by analyzing 16S rRNA sequencing data alongside other omics data. These AI-driven approaches facilitate the discovery of microbial signatures associated with ASD, which would be difficult to identify with traditional methods. These technologies could also be applied in personalized medicine, where multi-omics analysis has significant implications for ASD patients. By integrating individual microbiome profiles with genomic and metabolic data, researchers can identify unique biomarkers that stratify ASD subtypes and predict responses to interventions such as probiotics, dietary changes, or pharmaceuticals. This approach aligns with the goals of precision medicine by tailoring treatments based on each individual’s specific microbial and molecular profile. Advanced bioinformatics platforms, such as MicrobiomeAnalyst, QIIME 2, and emerging AI-driven systems like Deep Microbiome and MetaML, have shown potential in processing and visualizing multi-omics data. These tools enable cross-omics analysis and facilitate clinical translation by pinpointing potential therapeutic targets. Moving forward, the integration of AI with cloud computing and real-time data analytics will likely enhance the scalability and accessibility of multi-omics-based personalized medicine for ASD.

### 8.1. Advances in Bioinformatics and AI for MGB-Targeted Clinical Trials

Recent advances in bioinformatics and AI have significantly enhanced the analysis of complex multi-omics data in clinical trials targeting neuroinflammation and neurodegeneration in ASD. Probiotic and FMT interventions have shown promise in modulating the gut microbiota to reduce neuroinflammatory markers in ASD patients. AI-driven computational biology tools, including machine learning algorithms, enable researchers to analyze vast datasets, identifying microbial patterns and predicting patient responses to these interventions with higher precision. For instance, machine learning models can stratify ASD patients based on their unique microbiome profiles, tailoring probiotic treatments that are more likely to restore microbial balance and reduce neuroinflammation [100]. These advances support the development of personalized microbiome-based therapies that target the MGB axis, paving the way for precision medicine in ASD management.

### 8.2. Future Directions

The analysis of the microbiome using 16S rRNA sequencing provides foundational insights into microbial composition and diversity in individuals with ASD. However, it captures only a partial view of the microbiome’s complexity, as it lacks functional insight into how microbial communities affect the host. To achieve a more comprehensive understanding, complementary techniques such as metagenomics, transcriptomics, and metabolomics are essential. Metagenomics enables whole-genome sequencing of all organisms present—including bacteria, viruses, fungi, and archaea—thereby providing a more complete taxonomic and functional profile. Transcriptomics measures gene expression levels, offering a view of active microbial functions, while metabolomics profiles the metabolites produced by the microbiota, directly impacting host physiology and potentially linking to ASD-related pathways. Integrating these multi-omics approaches, although challenging, is crucial for advancing ASD research. Moreover, AI-enhanced bioinformatics tools can help researchers navigate and analyze complex datasets, fostering precision medicine in ASD and supporting the development of tailored therapeutic strategies. This multi-omics approach holds promise for unraveling the precise mechanisms behind microbiota-host interactions and identifying targeted interventions that address microbial imbalances unique to each patient.

## 9. Conclusions

Our review highlights consistent evidence of microbial dysbiosis in both gut and oral microbiota associated with ASD, showing marked differences from healthy controls. These findings emphasize the potential role of microbiota in modulating ASD symptoms, particularly gastrointestinal and behavioral manifestations. While 16S rRNA sequencing has provided critical baseline knowledge, continued research, including clinical trials and expanded multi-omics approaches, is necessary to confirm these findings and refine targeted therapeutic interventions. The identification of biomarkers (Section 7.4) not only aids in diagnosis but also supports the development of personalized treatments, targeting specific pathways. These advancements in biomarkers discovery and analysis tools are paving the way for personalized medicine in ASD. By integrating microbiome analysis with clinical practice, biomarkers, innovative bioinformatic tools, and AI, we move closer to understanding ASD’s underlying microbial imbalances and developing effective, personalized treatments.

## Figures and Tables

**Table 1:** Summary of significant clinical studies exploring the microbiota-gut-brain axis in ASD through 16S rRNA sequencing diagnostics. The table highlights microbial profiles associated with ASD symptomatology, including specific taxa such as *Prevotella*, *Bacteroides*, and *Clostridium*, and their correlation with behavioral and gastrointestinal symptoms.

Study	Study Population	Results	Clinical Correlations
Zuffa S. et al., 2023 [49]	60 children (30 ASD, 30 controls)	Increased *Prevotella* Decreased *Bifidobacterium*	Associated with greater severity of behavioral symptoms
Kang et al., 2017 [51]	18 children (18 ASD)	Increased *Clostridium* Reduced total microbial diversity	Related with gastrointestinal and behavioral symptoms
Strati et al., 2018 [52]	40 children (20 ASD, 20 controls)	Increased *Ruminococcus* Decreased *Bacteroides*	Associated with dysbiosis and gastrointestinal symptoms
Coretti et al., 2017 [53]	39 children (27 ASD, 12 controls)	Increased in *Lactobacillus* and *Clostridium* Decreased in *Bifidobacterium*	Related with gastrointestinal symptoms and behavioral changes
Liu et al., 2019 [28]	90 children (60 ASD, 30 controls)	Increased *Sutterella* Decreased *Prevotella*	Related with gut dysbiosis and inflammation

## List of Abbreviations

ANS	Autonomic Nervous System
AI	Artificial intelligence
ASD	Autism spectrum disorder
BBB	Blood-brain barrier
cfRNA	circulating free RNA
CNS	Central Nervous System
ENS	Enteric Nervous System
EV	extracellular vesicles
FMT	Fecal microbiota transplantation
FOS	Fructo-oligosaccharides
GABA	Gamma-aminobutyric acid
GFCF	Gluten-free, casein-free
GOS	Galacto-oligosaccharides
IBD	Inflammatory bowel diseases
IL-6	Interleukin-6
LPS	Lipopolysaccharides
miRNA	microRNA
MGB	Microbiota-gut-brain
ML	machine learning
MTT	Microbiota transfer therapy
NGS	Next Generation Sequencing
rRNA	Ribosomal RNA
SCFAs	Short-chain fatty acids
TNF-α	Tumor necrosis factor-alpha
